# Graphene oxide nanofilm and the addition of l-glutamine can promote development of embryonic muscle cells

**DOI:** 10.1186/s12951-020-00636-z

**Published:** 2020-05-15

**Authors:** Marlena Zielińska-Górska, Anna Hotowy, Mateusz Wierzbicki, Jaśmina Bałaban, Malwina Sosnowska, Sławomir Jaworski, Barbara Strojny, André Chwalibog, Ewa Sawosz

**Affiliations:** 1 Department of Nanobiotechnology and Experimental Ecology, Institute of Biology, Warsaw University of Life Sciences, 02-787 Warsaw, Poland; 2 Department of Veterinary and Animal Sciences, University of Copenhagen, 1870 Frederiksberg, Denmark

**Keywords:** Graphene oxide, l-glutamine, Chicken, Embryo, Muscle, Myogenesis, In vitro

## Abstract

**Background:**

Formation of muscular pseudo-tissue depends on muscle precursor cells, the extracellular matrix (ECM)-mimicking structure and factors stimulating cell differentiation. These three things cooperate and can create a tissue-like structure, however, their interrelationships are relatively unknown. The objective was to study the interaction between surface properties, culture medium composition and heterogeneous cell culture. We would like to demonstrate that changing the surface properties by coating with graphene oxide nanofilm (nGO) can affect cell behaviour and especially their need for the key amino acid l-glutamine (L-Glu).

**Results:**

Chicken embryo muscle cells and their precursors, cultured in vitro, were used as the experimental model. The mesenchymal stem cell, collected from the hind limb of the chicken embryo at day 8 were divided into 4 groups; the control group and groups treated with nGO, L-Glu and nGO supplied with L-Glu (nGOxL-Glu). The roughness of the surface of the plastic plate covered with nGO was much lower than a standard plate. The test of nGO biocompatibility demonstrated that the cells were willing to settle on the nGO without any toxic effects. Moreover, nGO by increasing hydrophilicity and reducing roughness and presumably through chemical bonds available on the GO surface stimulated the colonisation of primary stromal cells that promote embryonic satellite cells. The viability significantly increased in cells cultured on nGOxL-Glu. Observations of cell morphology showed that the most mature state of myogenesis was characteristic for the group nGOxL-Glu. This result was confirmed by increasing the expression of *MYF5* genes at mRNA and protein levels. nGO also increased the expression of *MYF5* and also very strongly the expression of *PAX7* at mRNA and protein levels. However, when analysing the expression of *PAX7*, a positive link was observed between the nGO surface and the addition of L-Glu.

**Conclusions:**

The use of nGO and L-Glu supplement may improve myogenesis and also the myogenic potential of myocytes and their precursors by promoting the formation of satellite cells. Studies have, for the first time, demonstrated positive cooperation between surface properties nGO and L-Glu supplementation to the culture medium regarding the myogenic potential of cells involved in muscle formation.
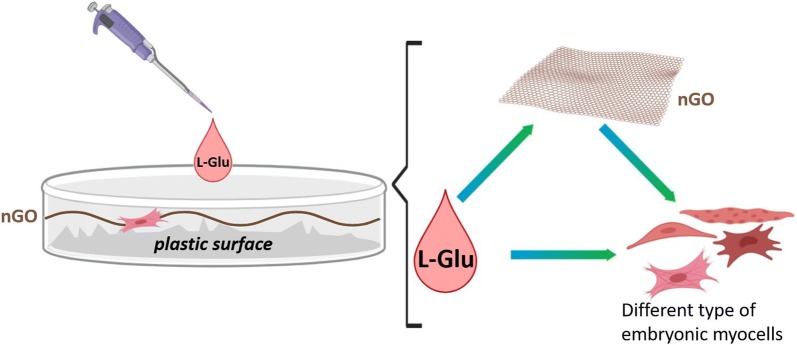

## Background

The cultivation of muscle tissue from muscle precursor cells, to obtain an autologous implant muscle tissue model for research or meat culture, requires optimisation of three key factors: cells, extracellular matrix (ECM)-mimicking structure and factors activating the process of cell differentiation and tissue maturation. All these factors can cooperate, changing the behaviour of cells and their fate. Furthermore, by secreting various factors, the cells can modify the interaction of the ECM and the surrounding molecules; therefore, the whole orchestra is responsible for creating a tissue-like structure. Interrelationships between cells, ECM-mimicking structure and the surrounding medium are relatively unknown. The purpose of our research was to show that changing surface properties can modify cell behaviour, especially their need for key amino acids.

Glutamine is the most abundant amino acid in the body. In skeletal muscle, which is the largest reservoir and producer of glutamine, it constitutes 50–60% of the free amino acid pool [1, 2]. l-glutamic acid, in addition to its function in protein structure, is involved in four key reactions of protein and energy metabolism. It is a permanent source of the amino group for the synthesis of endogenous amino acids, forming glutamine by binding the potentially toxic group NH_2_, a substrate for the synthesis of purines and pyrimidines and also a source of the carbon skeleton for gluconeogenesis [2–4].

The anabolic and detoxifying nature of glutamine activity determines its pro-proliferative nature [4]. It is also involved in the transport of an amino group between cells, which promotes tissue development. In sport, l-glutamine (L-Glu) is a commonly used supplement, that stimulates the metabolism of muscle tissue, however, knowledge on the antifatigue potential of glutamine is still not sufficient [2].

The physiological role of glutamine metabolism during embryonic development is not fully known [5], especially, considering the proliferation, differentiation and maturation of muscle tissue, the role of glutamine appears to be crucial. Undoubtedly, glutamine supports therapy reducing the degradation of muscle tissue, caused by inflammation, partly by satellite cell activation, especially after damaging exercise [6, 7]. Interestingly, experiments with the C2C12 myoblasts’ cell line, have shown that L-Glu improves skeletal muscle cell differentiation and prevents myotube atrophy after cytokine (TNF-α) via regulation of p38 MAPK [7]. Moreover, transaminase-dependent α-ketoglutarate production from glutamine is critical for the proliferation and differentiation of skeletal satellite cells [5].

The allotrope of carbon–graphene is a two-dimensional nanomaterial composed of carbon atoms in an sp2 hybrid orbital hexagonal honeycomb crystal lattice [8]. The structure of the graphene film also allows for easy attachment of various molecules to it, which gives the opportunity to modify its surface in a desired way [9]. Graphene materials, including graphene oxide (GO), seem to be the ideal materials for cell and tissue culture and tissue engineering applications; with a nano thin surface, exposing biologically friendly functional groups on its surface and being uniquely elastic, corrugated and durable, it may constitute a key element of artificial ECM [10]. GO scaffolds may be considered to produce different tissue-like structures such as blood vessels, skin, connective tissue and skeletal muscle, by providing an optimised microenvironment [11]. In experiments with human adipose stem cells (hASCs), researchers have observed that GO films are an efficient platform for hASCs cultures, consequently, GO may be used for designing and manipulating scaffold for stem cells, but also for tissue engineering applications [12]. Ku and Park [13] proved that GO, and reduced GO (rGO), supported adhesion and proliferation of cells of mouse myoblasts (C2C12), however, GO more significantly stimulated myogenic differentiation. Moreover, they suggested that the surface roughness and surface oxygen content influences the adsorption of serum proteins and may increase the myogenic potential of GO. In other in vitro studies with C2C12 muscle cells, it was found that GO, added to the medium, improved murine myoblasts differentiation cultured on Arg–Gly–Asp tripeptide/polylactic-co-glycolic acid nanofibre matrices. However, GO was cytotoxic to C2C12 cells at the concentrations ≥ 100 μg/mL, hence the authors used GO concentration at a low level, 10 μg/mL [14]. Other experiments, demonstrated that electrospun GO**–**poly(ε-caprolactone) fibrous scaffolds improved adhesion, proliferation and induced multinucleated myotube formation in human cord blood mesenchymal stem cells derived skeletal myoblast [15]. Also, research has demonstrated that composite conductive nanofibres of polyaniline and polyacrylonitrile with graphite and GO, prepared by an electrospinning process, were biocompatible to muscle satellite cells [16]; they suggested that the conductivity and stiffness of composite nanofibres plays a key role in the proliferation and differentiation of satellite cells.

There are not many publications on the formation of muscle fibres on the GO platform that would allow the elimination of additional compounds from a potential implant. Ahadian et al. [17] investigated ultrathin GO and rGO-based films to obtain pseudo-muscle tissue; because of the bigger conductivity, rGO was a perfect platform to obtain the free-standing muscle myofibres, exhibiting contractility upon applying the electric field. However, rGO is much more hydrophobic, which limits its usage as an implant.

Our previous experiments on rats revealed that GO injected intraperitoneally has low toxicity and is strongly agglomerated, and then gradually is removed from the body [18]. Trends in agglomeration, under the influence of proteins present in the body, as well as the very small amount of GO used as a niche, determines its unique application. However, increased proliferation and differentiation, as well as muscle fibre formation, are associated with the inevitable higher demand for protein and energy. Depending on the direction of metabolism, glutamic acid can be a source of both energy and protein, and what is more, by removing unnecessary ammonia, it also plays a detoxifying role.

In the present research, we have hypothesised that changing the surface properties by covering it with biocompatible graphene oxide nanofilm (nGO) and adding L-Glu as a universal source of energy and/or protein, can change the behaviour of cells in heterogeneous culture of muscle cells and their precursors.

## Results

### GO nanofilm surface and biocompatibility characterisation

**Fig. 1:**
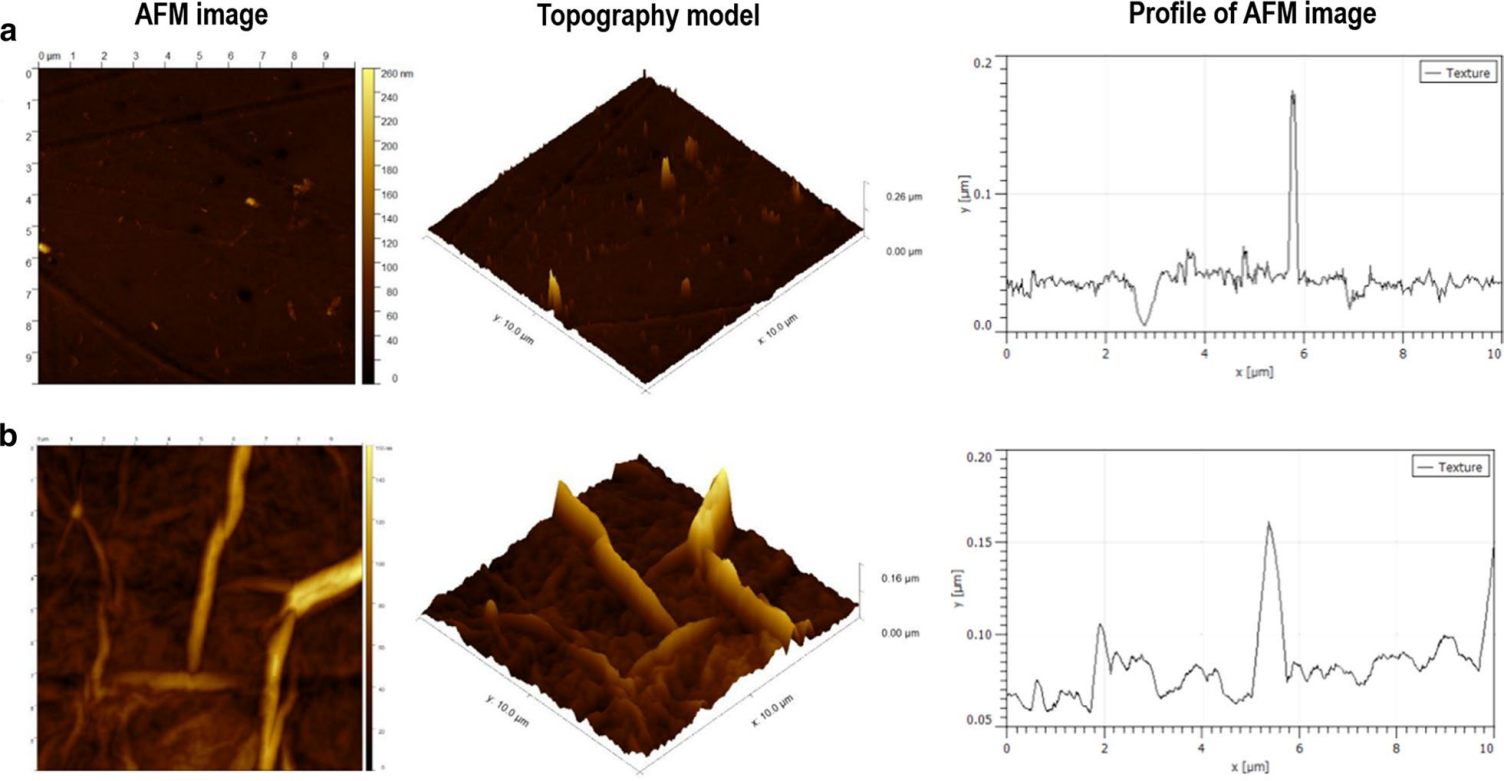
Graphene oxide nanofilm surface characterisation compared to clear culture plastic dish, evaluated with Atomic Force Microscopy analysis (AFM). Images present a topography model and profile of image of the clear surface of the plastic culture dish (**a**) and surface of a graphene oxide nanofilm covered culture plate (**b**)

As demonstrated by Atomic Force Microscopy (AFM) analysis, there was a significant difference between the surface of the plastic bottom of a standard culture plate and plate covered with nGO. In general, the surface of the nanofilm was flatter, less porous, less diverse and gentler in comparison to the plastic plate (Fig. 1a, b). The results concerning the roughness parameters of both surfaces are presented in Table 1. All parameters of the nGO surface were much smaller than for the plastic dish. The average roughness of the nGO was four times less than the average roughness of the culture dish clear surface, and the root mean square roughness was more than five times greater for plastic than for GO surface. Also, the maximum height of the roughness, as well as average maximum roughness valley depth, were higher for plastic than for nGO surface, which proved that the plastic surface had a greater sharpness than the GO coated surface. In summary, the plastic cover was the cause of the surface levelling and softening its roughness.

**Table 1:** The comparison of the surface roughness between the plastic culture dish and the same plate covered by graphene oxide nanofilm (nGO), measured by atomic force microscopy

The parameters of roughness (nm)	Surface	
	Plastic plate	nGO
Maximum height of the roughness	155 ± 37.4	20.45 ± 7.33
Maximum roughness valley depth	57.8 ± 16.11	13.7 ± 5.42
Average roughness	7.2 ± 2.62	1.8 ± 0.43
Root mean square roughness	13.3 ± 3.73	2.4 ± 0.71

Mean values and ± standard deviation

**Fig. 2:**
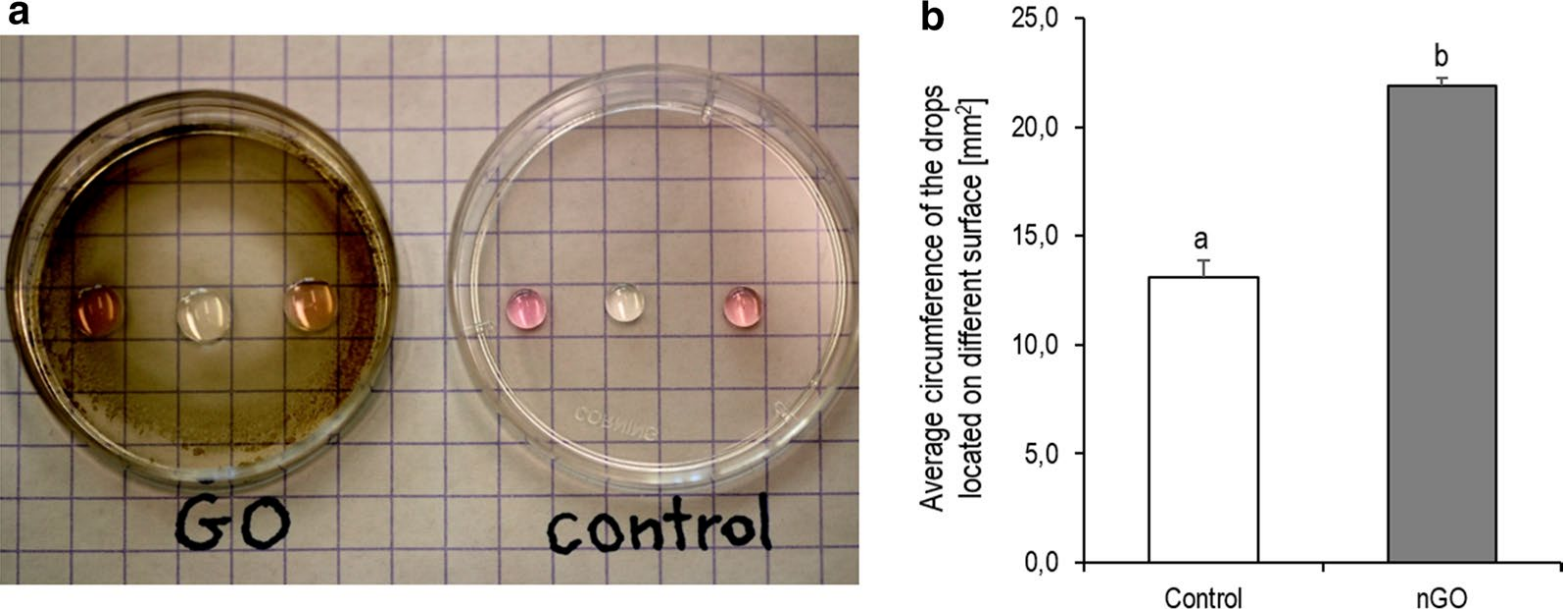
**a** The digital visualisation of the hydrophilicity properties of graphene oxide nanofilm (nGO) surface in relation to the plastic plate surface. **b** The comparison of the average circumference of the drops (water and Dulbecco’s modified Eagle medium; DMEM) located on the clear surface of the culture plastic dish (control) and on the surface coated with nGO. The error bars represent standard error of mean (*n* = 6 per group). Different letters (a–b) above the columns indicate statistically significant differences (*p* ≤ 0.05) analysed by t-test. Measurements were performed using ImageJ software

To evaluate the hydrophilicity of nGO, the circumference of the drops were measured. As shown in Fig. 2b, the average circumference of drops on nGO was significantly higher compared to the control (+ 67% increased, *p* ≤ 0.05). This result indicated the greater hydrophilicity properties of nGO in relation to the plastic plate surface.

**Fig. 3:**
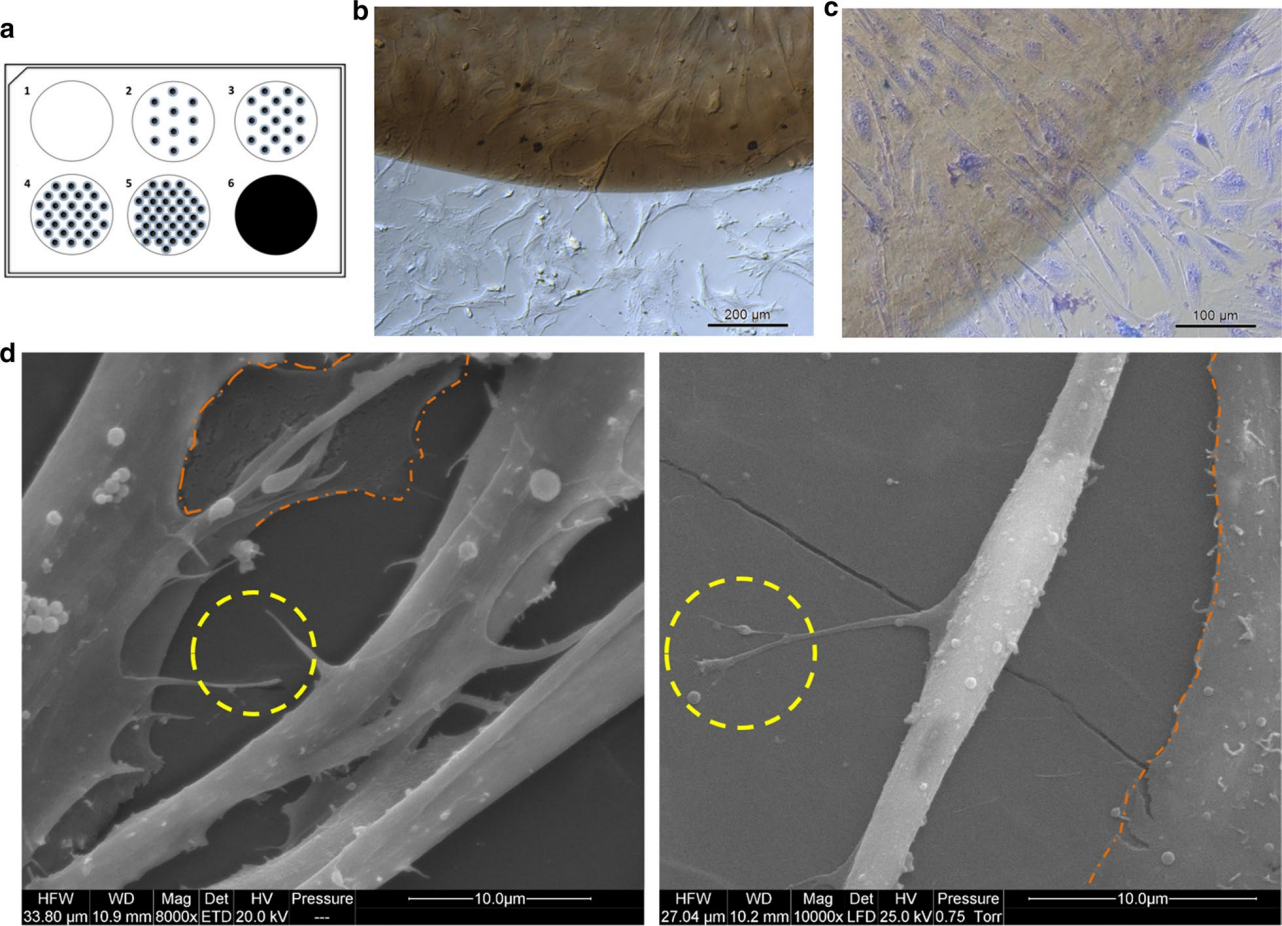
The visualisation of graphene oxide nanifilm (nGO) biocompatibility with mesenchymal stem cells after 48 h of incubation (magnification ×10 and ×20) (**b**, **c**) or after 5 days of incubation (**c**; magnification ×8000 and ×10,000). **a** The pattern of covering the surface of the culture plate with nGO: Well 1 = control (without nGO); wells 2 to 5 = gradually increasing the number of nGO dots; well 6 = complete surface coverage with nGO. **b** Live imaging observation of cells migration towards nGO dots. **c** Hematoxylin and eosin staining of cells growing on nGO dot. **d** Scanning electron microscopy visualisation of primary muscle fibre grown on nGO; the yellow rings indicate the cell insert directly connected to nGO; the orange surround shows the primary stromal cells base layer

The simple test of nGO biocompatibility was also performed by analysing the colonisation of nGO dot surface by cells (the pattern of nGO dots niche at this experiment is presented at Fig. 3a). In Fig. 3b, c, it can be observed that mesenchymal stem cells after 48 h of incubation, migrated and grew on the nGO dots. Numerous cells were observed both around the dot, in its centre and on the outskirts of the nGO; meaning that the muscle cells and their precursors were willing to settle the nGO niche, and nGO at the tested concentration had no toxic effect on them. This observation documents that nGO is a friendly plane for the observed cells, which was also supported by TEM visualisation of formatted primary muscle fibres forming insets directly to nGO and not to the primary stromal cells base layer, after 5 days of incubation (Fig. 3d).

### Cell morphology

**Fig. 4:**
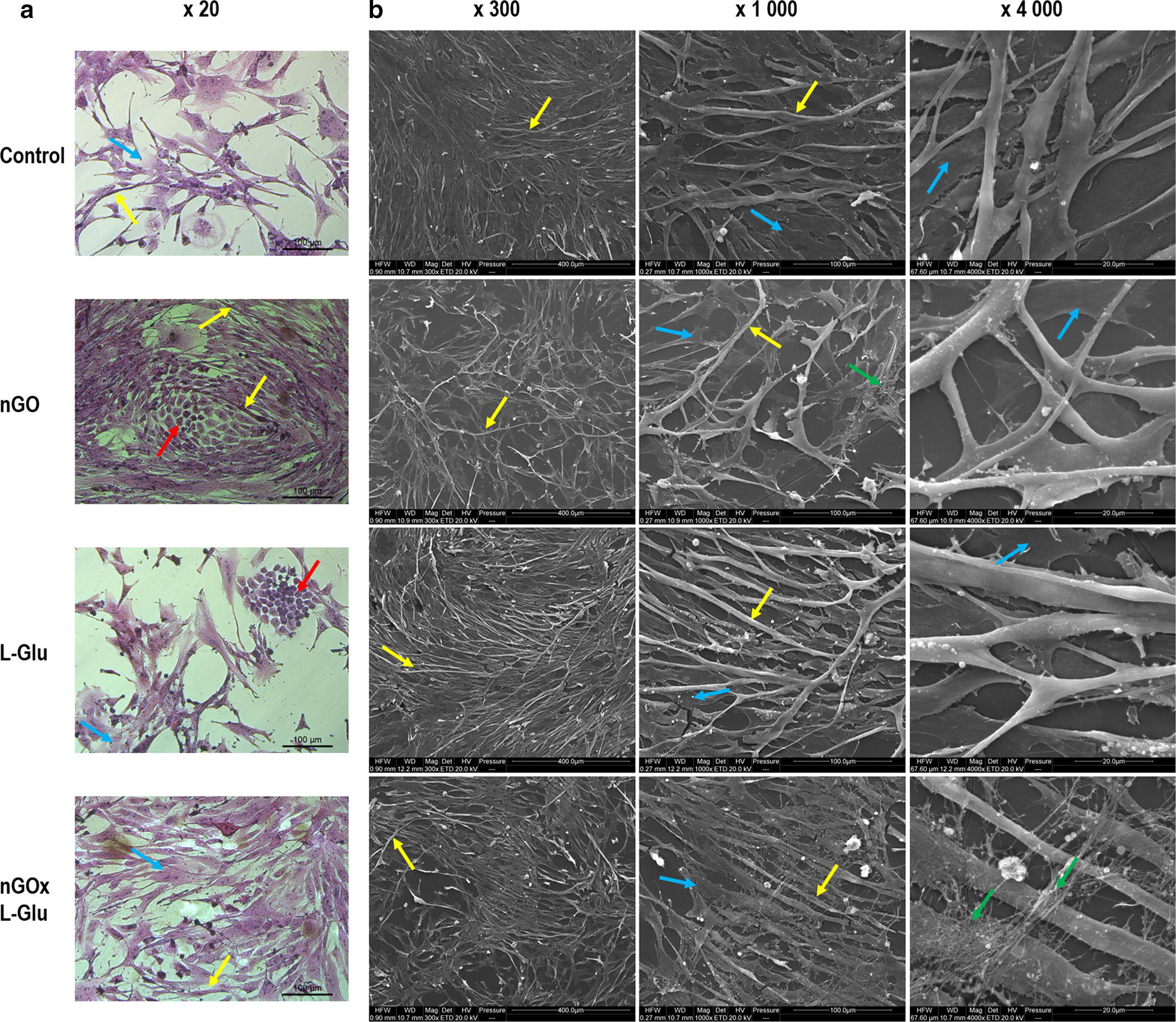
Cell morphology evaluated by optical microscopy (**a**) and scanning electron microscopy (**b**). **a** The images show cells cultured 5 days and hematoxylin and eosin stained; the red arrow indicates satellite cells, the yellow arrow indicates myofibres, the blue arrow indicates primary stromal cells; **b** the images show the control group cells cultured 5 days and the cells cultured 5 days on graphene oxide nanofilm (nGO), l-glutamine (L-Glu), and nGO with L-Glu supplementation (nGOxL-Glu); primary muscle fibres (yellow arrows), collagen fibres (green arrows), primary stromal cells (blue arrows)

The cells, observed under a microscope, were a collection of different types of cells taken from the muscle of an 8-day-old chicken embryo, and thus constituted a mixture of different cells, mostly constituting precursor cells of striated muscles. After 5 days of incubation, the cells formed two main groups (Fig. 4). The first type of cell showed foetal stem cells appearance, cells were round and elliptic, with dense cytoplasm, one nucleus and cytoplasmic protrusions. The second group were elongated cells that differentiated into myocytes and longer primary myofibres, having more than one nucleus. Consequently, this group of cells can be divided into cells in the early phase of differentiation, characterised by a developed surface, frayed shape and a large number of filopodia, and cells in the further phase of differentiation, forming multinucleated thin and long fibres.

Cells from the control group represented all these forms of cells (Fig. 4a). In turn, in the cell population maintained with the addition of L-Glu, there was a large number of stem cells, which formed large clusters (Fig. 4a; red arrows). In the population of cells incubated on nGO, a greater number of cells were observed differentiating into primary muscle fibres (Fig. 4a, yellow arrows). Interestingly, cells grown on the nGO surface, and receiving L-Glu in the medium, formed both denser stem cell clusters as well as more cells characteristic of differentiating myocytes were seen.

This observation was confirmed by SEM images (Fig. 4b), allowing a more detailed picture. Observation of cells at higher magnification confirmed that they formed two layers of cells, differing not only in morphology but also in the location. The first, primary stromal cells layer (Fig. 4b, blue arrows), tightly adhering to the culture dish, formed a kind of base layer. They were very flat, elliptic cells of various shapes, ranging from round to slightly elongated, and even branched. These cells created a dense plane, strongly adhered to the bottom of culture flask (or nGO) layer. This layer of cells was in contact with differentiating muscle precursor cells. The second layer of cells was muscle cells and primary fibers. The formation of muscle fibres ‘sprouting’ from the cell layer adjacent to the ground was clearly visible. Furthermore, the muscle primary fibres that were formed were anchored in this cell layer with cytoplasmic protrusions. Differentiating muscle fibres maintained contact with the base of primary stromal cells layer. Compared to the control group, the primary stromal cell layer appeared to be the densest in the L-Glu group. Observation of differentiating muscle fibres showed that the longest, thin and parallel fibres were in the L-Glu group. In the nGO group, muscle fibres and their precursors were highly branched, which indicated their intense fusion. They were arranged less parallel and more chaotically, and were stronger and thicker. The best-developed fibres were observed in the nGOxL-Glu group, moreover, the cells were surrounded by a clearly visible layer of collagen fibres. Stem cells were also visible in all groups, however, in groups with nGO, more dividing foetal stem cells can be seen (Fig. 4b).

### Cell differentiation

**Fig. 5:**
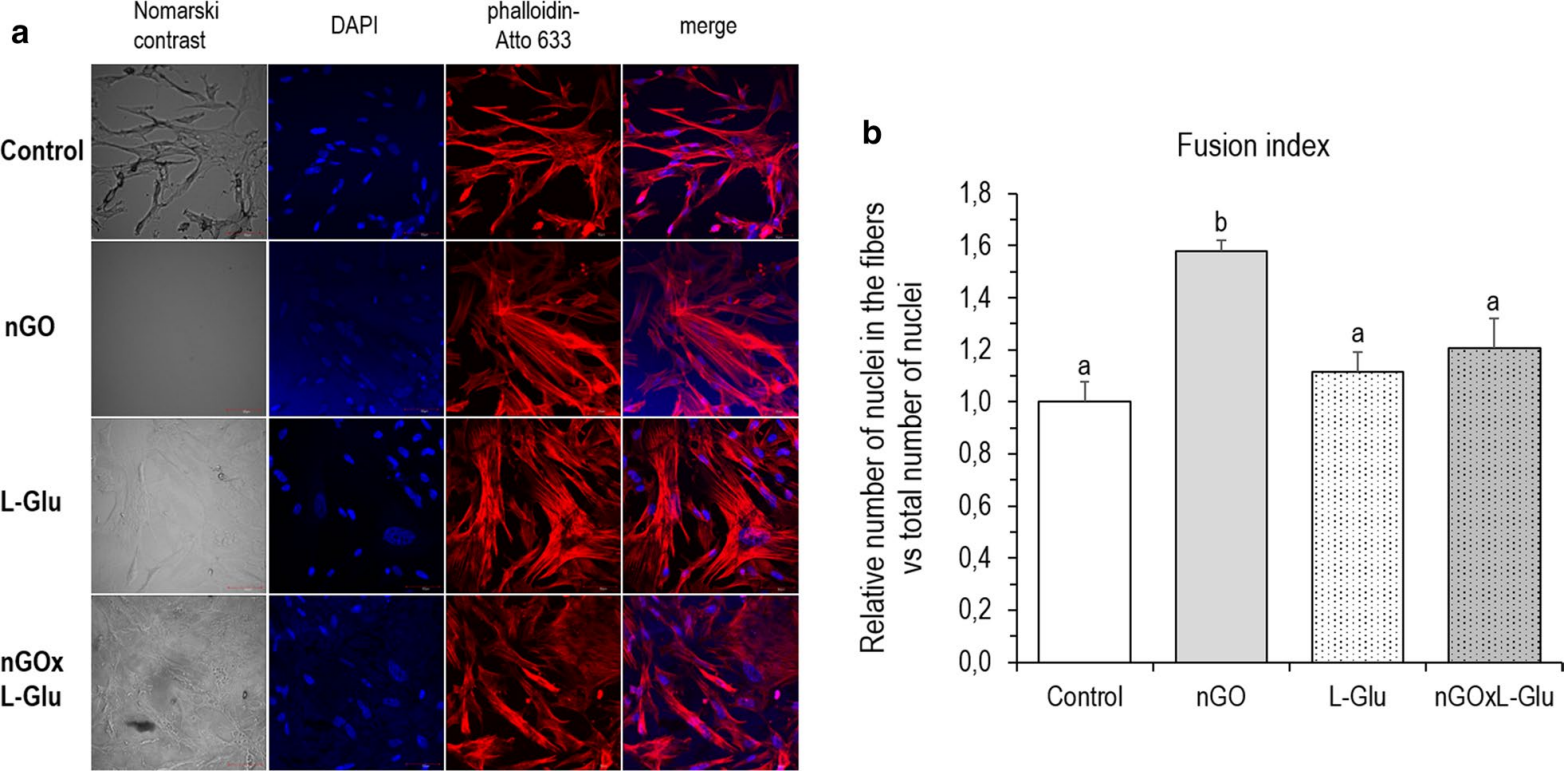
Differentiation status of the chicken embryo muscle control cells and treated with graphene oxide nanofilm (nGO), l-glutamine (L-Glu), and on nGO with L-Glu supplementation (nGOxL-Glu). **a** Confocal microscopy visualisation images (×40 magnification): grey = Nomarski interference contrast, blue = 4′,6-diamidino-2-phenylindole (DAPI) nuclei staining, red = actin filaments with phalloidin-Atto 633 staining; **b** quantitative analysis of the fusion index of the differentiating cells. The error bars represent standard error of mean (*n* = 8 per group). Different letters (a–b) above the columns indicate statistically significant differences (*p* ≤ 0.05) analysed by Tukey’s HSD test

To verify the differentiation status of the primary culture of muscle cells after 5 days of incubation with investigated factors (L-Glu and nGO scaffolds), the fusion index was determined based on fluorescence staining. The results are presented in Fig. 5. The relative fusion index significantly increased in group culture on nGO, when compared to the control and other groups. There were no differences after L-Glu action.

### Cell viability

**Fig. 6:**
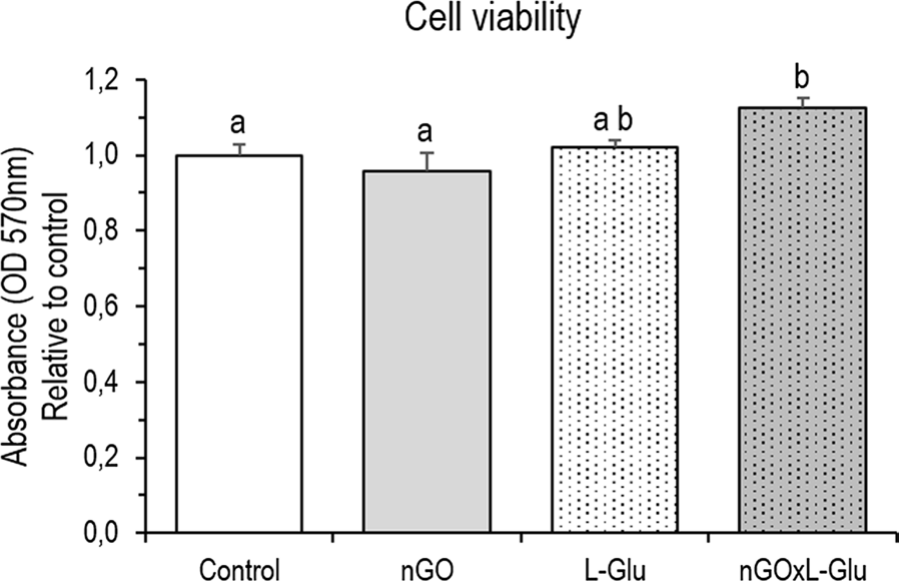
Mesenchymal stem cells viability measured by mitochondrial activity depletion in the control group and cells treated with graphene oxide nanofilm (nGO), l-glutamine (L-Glu) and nGO with L-Glu supplementation (nGOxL-Glu). MTT assay was performed after 48 h of primary culture. The error bars represent standard error of mean (*n* = 6 per group). Different letters (a–b) above the columns indicate statistically significant differences (*p* ≤ 0.05) analysed by Tukey’s HSD test

To compare the effect of nGO, L-Glu additive and the use of both nGO and L-Glu on culture cells viability, the MTT test was performed. Measurement of colour intensity after conversion of MTT into formazan by viable cells with an active metabolism showed a significant increase of viability of cells after nGOxL-Glu treatment compared to the control and nGO groups. It should also be noted that no significant differences in viability were observed between cells from the control group and those that colonised nGO surface, that is, no toxic effects were been demonstrated. The results are shown in Fig. 6.

### Relative gene expression at the mRNA level

**Fig. 7:**
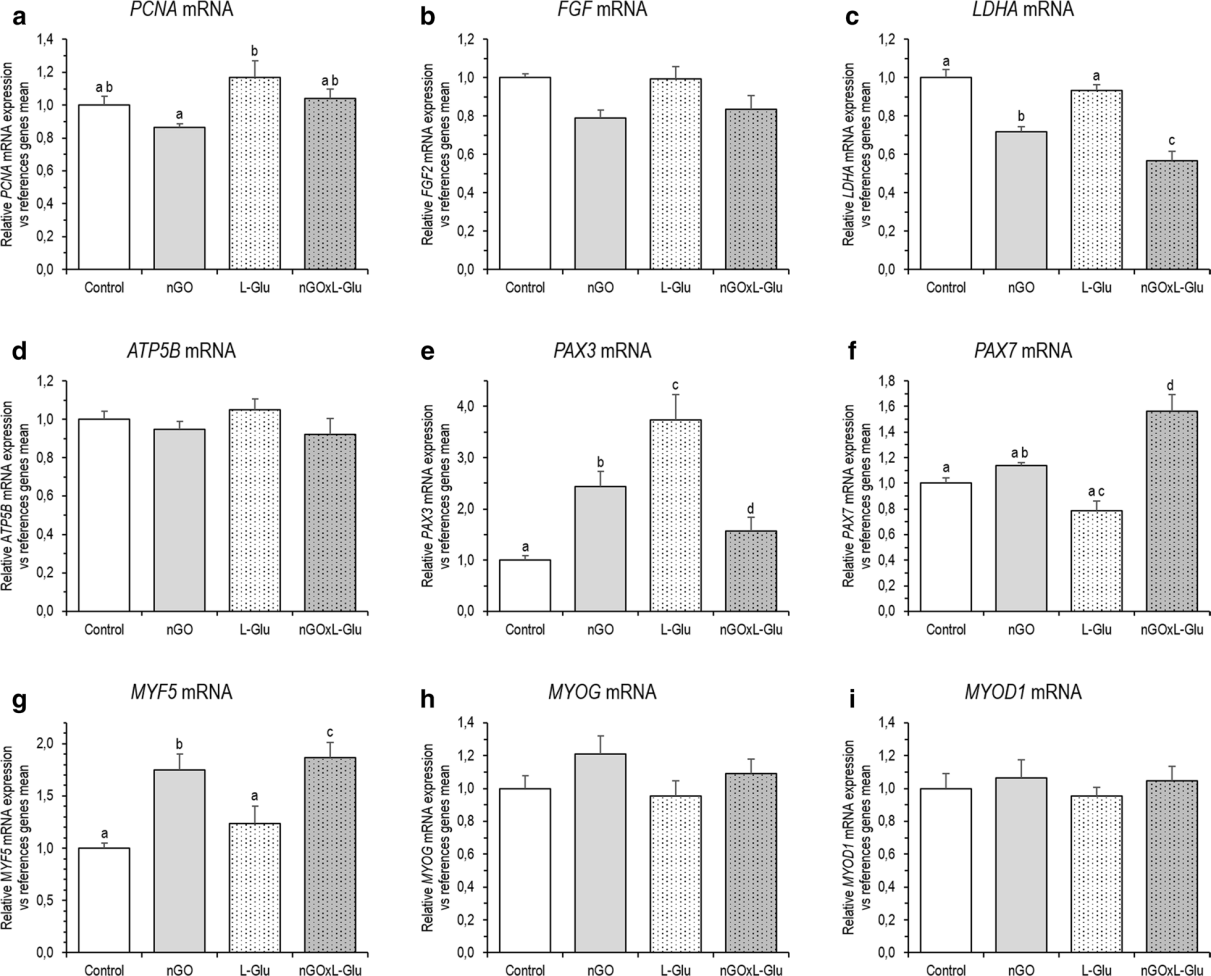
mRNA expression of genes related to proliferation (**a**–**d**), and muscle cells differentiation (**e**–**i**) in the muscle progenitor cells from the chicken embryo after 5 days of primary culture at the mRNA level, determined according to the real-time qPCR method. The figure shows the results for the control group and groups treated with nGO, L-Glu, and nGO with addition of the L-Glu (nGOxL-Glu). The results represent a relative expression of the respective target gene vs. reference genes mean. The error bars represent standard error of mean (*n* = 6 per group). Different letters (a–d) above the columns indicate statistically significant differences (*p* ≤ 0.05) analysed by Tukey’s HSD test

According to qPCR analysis, after 5 days of primary cell culture no effects of nGO, L-Glu as well as both of these factors compared to control on proliferating cell nuclear antigen (*PCNA*) expression at mRNA level were found. However, an increase in PCNA expression was observed in the L-Glu group compared to nGO cells (Fig. 7a). Also no effect on the expression of fibroblast growth factor 2 (*FGF2*) after nGO and L-Glu treatment was observed (Fig. 7b). Furthermore, all experimental factors reduced lactate dehydrogenase A (*LDHA*) expression associated with anaerobic oxidation. The use of L-Glu with nGO reduced the expression of *LDHA* mRNA to the greatest extent (Fig. 7c). The level of ATP synthase, H+ transporting, mitochondrial F1 complex, beta polypeptide (*ATP5B*) mRNA, involved in aerobic energy metabolism, however, was not changed (Fig. 7d).

In the experiment, the expression of genes involved in myogenesis, such as paired box 3 (*PAX3*), paired box 7 (*PAX7*), myogenic factor 5 (*MYF5*), myogenin (*MYOG*) and myogenic differentiation 1 (*MYOD1*) was analysed. *PAX3* expression at mRNA level in all groups increased compared to the control group (Fig. 7e), however, to the greatest extent under the influence of L-Glu and the least under the influence of the use of both factors (nGOxL-Glu). Covering the surface of the vessel with nGO also upregulated *PAX7*, however, the addition of L-Glu reduced the level of mRNA of this gene. Interestingly, the use of nGO surface and the addition of L-Glu strongly upregulated *PAX7* expression (Fig. 7f).

In turn, only in one case from three tested genes associated with the differentiation process, was the mRNA expression significantly upregulated by nGO. The expression of *MYF5* increased by 74% in comparison to control (Fig. 7g). No significant difference under the nGO influence was observed in *MYOG* and *MYOD1* mRNA expression (respectively Fig. 7h, i), however, a tendency to increase MYOG expression under the influence of nGO could be seen. The addition of l-glutamine did not affect the regulation of gene expression involved in muscle cell differentiation, and only *MYF5* was slightly upregulated. *MYF5* expression in cells cultured on nGO supplemented with L-Glu was similar to the nGO group.

### Relative protein expression

**Fig. 8:**
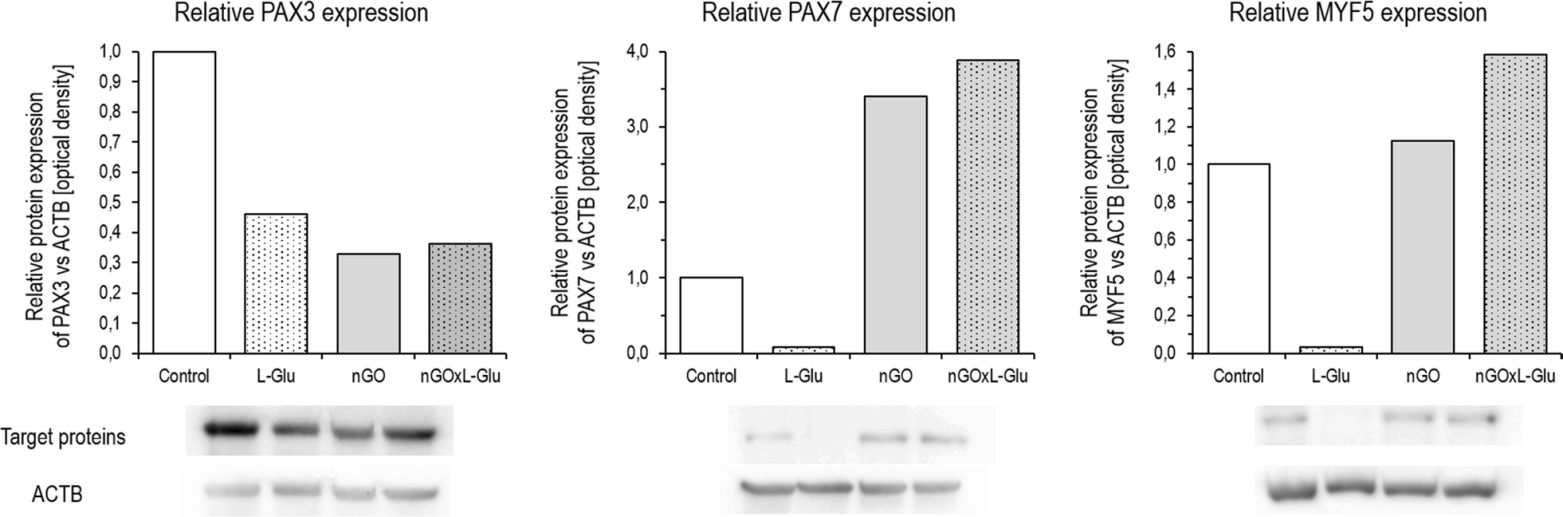
Protein expression in the muscle progenitor cells from the chicken embryo after 5 days of primary culture, determined according to the Western blot method. The figure shows the results for the control group and groups treated with l-glutamine (L-Glu), graphene oxide nanofilm (nGO) and nGO with addition of the L-Glu (nGOxL-Glu). The results represent a relative protein expression of the respective target proteins PAX3 (53 kDa), PAX7 (57 kDa) and MYF5 (28 kDa) vs. reference protein ACTB (43 kDa). Densitometric analysis of the scanned membranes was performed using ImageJ software

To determine the translational activity (protein expression) of chosen proteins related to differentiation, Western blot analysis was performed after 5 days of primary culture with tested factors. Incubation with L-Glu, comparing to control, strongly downregulated expression of all investigated proteins; PAX3, PAX7 and MYF5. In turn, nGO upregulated PAX7 and slightly MYF5 expression but simultaneously decreased PAX3 level. Interestingly, the introduction of nGO and the addition of L-Glu to the culture medium most, compared to all other groups, increased the expression of PAX7 and MYF5 (Fig. 8).

## Discussion

In classic terms, cell growth and differentiation depend on three basic things; ECM, signalling factors and the type and status of cells. However, usually, only in 3-D cultures is the multifunctional effect caused by an artificial ECM [19]. In vitro 2-D culture does not take into account the surface effect, limiting itself to a standard plastic culture vessel [20], although the surface of different culture plates may also vary significantly and also by operating in the nano dimension, the surface of the culture vessel can also be seen as a 3-D structure. For this reason, in the present research, we wanted to explain the impact of surface shaping/topography on the growth and differentiation of muscle cells and their precursors.

In vitro culture based on cell lines, allows very precise observation of specific mechanisms; however, their behaviour may differ significantly from the primary culture of cells [21]. Firstly, because of the loss of the natural heterogeneity of the tissue and also because of the multitude of signal factors sent and received by the cells, which, in this way, cooperate and modify each other [22]. Primary culture, however, creates difficulties because it is a dynamically differentiating cell community, but allows us to approach the real conditions of their growth and development.

The aim of the conducted experiments was to show that changing the properties of the surface, on which the cells are grown, also changes their behaviour. Above all, however, the effect caused by the interaction of the material surface (here nGO) can significantly change the requirement for energy/protein or selected nutritional or functional compounds. In our work, we showed for the first time the interaction between the influence of surface, modified by the nGO, and the effect of L-Glu added to the culture medium on the growth and differentiation of the population of foetal muscle cells and their precursors.

The nGO did not adversely affect cell viability. In our previous studies [23], we found that the nGO is not toxic for cells, taken from chicken embryo hind limb on the eighth day. Numerous experiments have been conducted about the toxicity and biocompatibility of GO in vitro, which indicate its harmful effects [24], including DNA damage [25, 26]. These toxic effects were mainly documented through in vivo studies, but also through in vitro experiments in which GO was administered to the culture medium. To summarise, in graphene oxide flake toxicity studies, it is found that the smaller the flakes and the greater the concentration in the culture fluid, the greater the toxicity [27]. However, it should be noted that in living organisms, muscle cells are not so much surrounded by fluid, but form a compact structure with the ECM (or other cells).

Less research has concerned the testing of toxicity of the GO coated surface. According to Wychowaniec et al. [27], use of GO as a surface-induced lower cytotoxicity toward human embryonic kidney cells and human neuroblastoma cells than GO used as a dispersion at concentrations of up to 200 μg/mL. In our experiment, with dots of GO, allowing visualisation of cell preferences for placement on the substrate of the film GO vs. plastic dishes, we showed that cells willingly populated areas with nGO. Moreover, there were no symptoms of nGO dot cell toxicity. The free choice of cells to colonise nGO areas showed a lack of toxicity of the surface covered with nGO; however, these results can only be related to the GO used in our research, as it is known that GO features may vary depending on many factors [28].

The mechanisms of nGO surface–cell interactions are very different and also depend on the cell model, and above all, on GO and its physical and chemical characteristics. A surface covered with a GO film, with the availability of oxygen groups on the surface, induced oxidative stress in human osteoblast cells as opposed to human gingiva fibroblast cells, however, this GO film was not significantly toxic for both cell types [29]. Nevertheless, oxygenated groups assist the GO hydrophilic nature, which increases GO solubility [30] and may favour cell colonisation. In studies on the biocompatibility of Si/GO surface using breast cancer cells, the authors even suggest hydrophobicity-driven cell growth [31]. The nGO used in our research was slightly more hydrophilic compared to the surface of the culture vessel; this could favour the colonisation of primary stromal cells with epithelial-like phenotype. Observations of morphology during the colonisation of the GO plane by cells showed that the first cell layer (primary stromal cells) adhered tightly, with a large surface of its body to the GO surface. In contrast, differentiating myocytes, as well as stem cells, are relatively poorly associated with the surface, but rather with primary stromal cells. Thus, it seems that the physicochemical features of the surface are directly crucial for the first layer of cells, but also, directly for differentiating myocytes and stem cells. However, the GO plane in the self-assembling process can be decorated with various organic molecules present in the culture medium [32], which can also determine the affinity of cells for GO. The resulting molecular pattern on the GO surface can determine the biointeraction of its surface with specific cell structures and consequently recognition [33].

Another important feature of the surface is its roughness, which may play a key role in cell attachment and proliferation [27]. In the present study, the surface coverage of the culture vessel softened sharp peaks and plaque cavities. Coating the surface of the culture dish with nGO alleviated surface roughness, although it did not lead to a complete reduction of the unevenness of the culture surface of the vessel. Studies by other authors on GO paper have demonstrated that different cells prefer different types of surface roughness. For example, human foetal osteoblastic cells showed optimal adhesion and spreading for the range between 5 and 15 nm [34], neurons had a strong adhesion for Ra about 25 nm [35] and macrophages adhered preferentially on a planar surface [36]. According to other authors, cells of the different cell lines (primary human endothelial cells, human epithelial cancer cell and mouse mesenchymal normal cell line) showed stable cell adhesion and proliferation on moderately rough substrates (10–45 nm) [37], although the roughness of the ultra-thin GO membranes, used among others as filters was 0.6–0.8 nm [28].

The possibility of forming graphene surfaces with specific morphology has been used in cell culture. Mouse myoblast cells (C2C12) inoculated on uniaxially crumpled graphene, became aligned and elongated at the single-cell level, as well the observation of the differentiation and maturation of myotubes compared to that on flat graphene [38]. In our research, we used the nGO surface, which was formed in the process of self-organisation (during drying), so it did not have an ordered surface, imitating the shape of developing fibres. Cells cultured on such nGO were also less ordered, they formed a more bushy structure, while in numerous places, the muscle cells were seen to be arranged perpendicularly in some places more than parallel. However, the group nGOxL-Glu cells formed a more ordered structure, arranged more in parallel. It seems that the first layer of primary stromal cells, supported by L-Glu, that directly cover the nGO reduced the surface effect, and in this case, the disordered roughness of the GO film. Jasim et al., [39] concluded that substrates based on GO papers are suitable biocompatible cellular structures for anchorage-dependent cell growth, especially because the adhesion of proteins, including growth factors, on a graphene surface was encouraged by interactions with π–π stacking [10]. The GO nanofilm, prepared from a 100 mg/L aqueous GO solution, probably formed a very thin film, as confirmed by both AFM results and TEM images. The film covered a thin mesh of rugged bottom of the plastic culture plate, creating a “hammock” effect for cells. Furthermore, the stretched GO flakes were more exposed on the surface of functional groups and π–π stacking to the interaction with L-Glu as well with cells. It can be hypothesised that a properly formed GO nanofilm, as delicate springing support on the one hand, and on the other hand a plane maximally opened to interactions with added L-Glu, created more favourable possibilities for the location of primary stromal cells.

During chicken embryogenesis, muscles of the limbs originate from the somite. Progenitor cells migrate to their final destination in the limb and then quickly start to differentiate by the activation of the muscle determination factors MYOD, MYOG and MRF4 [40]. Foetal myoblasts are most abundant from days 8 to 12 and then undergo massive differentiation at days 16 to 18 of embryogenesis [41]. Consequently, in our studies, the cells, taken from the embryo muscle at embryonic development stage 34 [42] and cultured in vitro, were a mixture of cells commencing intensive myogenesis, as confirmed by mRNA expression of genes involved in the development and differentiation of myogenic cells such as *MYF5*, *MYOD1* and *MYOG*, but also *PAX7* and *PAX3*. These cells, whose common feature is the origin of the dermomyotome [43] were a mixture of two key groups; muscle cells and their precursors, as well as satellite cells and their precursors, however, other cells such as primary stromal cells were also present in the heterogeneous culture. Cells of the dermomyotome showed expression of the *PAX3* and *PAX7*, and also a low level of *MYF5* expression [44]. The first muscle structure–myotome expressed MYOD, MYF5, played a role as markers of a terminal specification to the muscle lineage [45]. However, MYF5, in contrast to MYOD, may act in parallel with the PAX transcription factors [40, 46]. Despite the expression of genes that are markers of various stages of muscle maturation, the GO surface affected the change in expression of only specific genes. The increased number of multinucleated fibres, as well as the significant increase in *MYF5* expression and the tendencies towards increased *MYOG* expression at the mRNA level, indicate that the surface of nGO positively regulated the mechanisms of differentiation of muscle precursor cells. However, the presence of the cells expressing *MYOD1* was not increased by nGO. When considering the increasing number of cells with expression of *PAX3* and *PAX7*, in parallel with *MYF5,* it can be presumed that the effect of the nGO was more on the activation of myogenesis in the earlier phase of differentiation, or on the proliferation of the precursor of satellite cells. Nevertheless, gene expression at the mRNA level is not synonymous with gene expression at the protein level. By analysing protein expression, a very significant increase in PAX7 levels can be observed. This quite clearly indicates that the GO surface strongly promotes satellite cell development.

The addition of glutamine very significantly up-regulated PAX3, which may suggest a high demand for early forms of muscle and satellite cell precursors for energy or protein. However, under natural embryogenesis conditions, energy deficiency is observed rather than protein [47]. Nevertheless, administration to the chicken embryo L-Glu positively affects its further development, as well as development after hatching [48, 49]. In our experiments, the addition of an energy source in the form of L-Glu also reduced the need for anaerobic glucose degradation, illustrated by *LDHA* expression down-regulation. Interestingly, the use of GO surface with simultaneous administration of L-Glu to the medium increased cell viability, which further increased PAX7 expression, both at mRNA and protein levels.

This positive reaction, resulting from the modification of the culture surface by nGO, with the simultaneous increase of L-Glu level in the culture medium, found its expression both in increasing cell viability compared to other groups as well as reducing anaerobic glucose degradation (reducing *LDHA* expression), and above all in the up-regulation of PAX7 at the mRNA and protein level. Consequently, the use of nGO and L-Glu supplements may improve myogenesis and the myogenic potential of myocytes and their precursors by promoting the formation of satellite cells.

## Conclusions

The conducted experiments confirmed the lack of nGO toxicity and the possibility of using it to mimic ECM. Furthermore, we have shown that in the primary, heterogeneous culture, the used factors can modify the behaviour of one group of cells, and only those cells can decide about the behaviour of the other cells. The interaction between the layer of primary stromal cells and embryonic muscle cells and their precursors may determine the behaviour of the latter. Thus, increasing hydrophilicity and reducing roughness and presumably through chemical bonds available on the GO surface, by coating the in vitro culture vessel with nGO stimulated the colonisation of primary stromal cells that promoted embryonic satellite cells. Above all, however, we have documented that a change in surface properties, by changing the behaviour of cells and their activity, must be parallel to the change in the nutrients available to the cells. The mechanisms of this phenomenon remain unclear, but it seems that both GO and L-Glu supplementation should be considered during the in vitro muscle culture process. In addition, we suspect that this mechanism could be used in muscle cells from other animal species.

## Methods

### Experimental factors characterisation

**Fig. 9:**
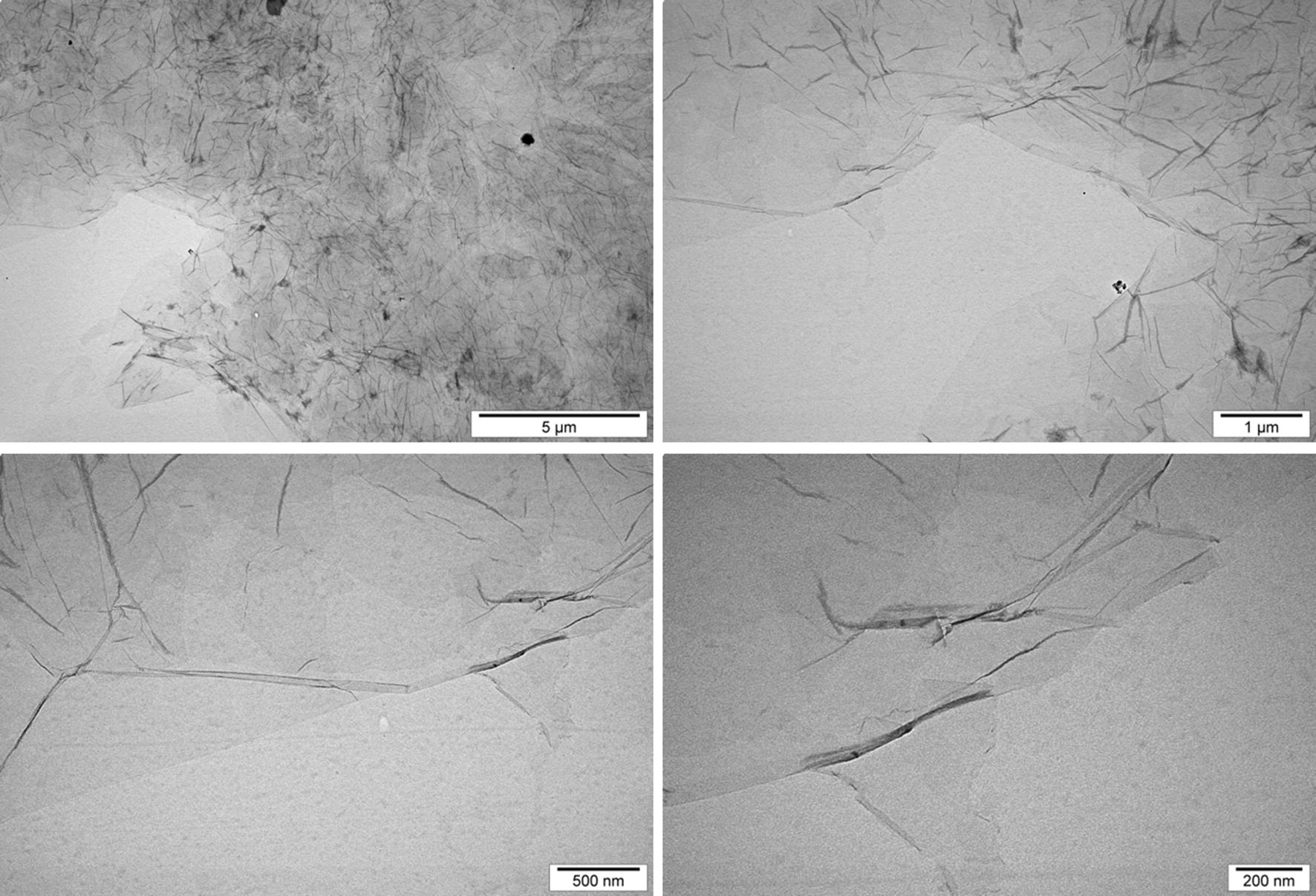
Transmission electron microscopy images of graphene oxide flakes

GO flakes, as an aqueous solution of 4 mg/mL concentration was obtained from NanoPoz (Poznan, Poland), and was produced by a modified Hummers method with a concentration of oxygen 36%. GO solution was suspended in ultra-pure water to give a concentration of 100 mg/L for further measurements. The GO aqueous solution was characterised by high stability, no tendency to agglomerate and its zeta potential was − 21.6 mV (ZetaSizer Nano ZS model ZEN3500, Malvern Instruments, Malvern, UK). The shape and size of the GO films were examined using transmission electron microscopy (TEM) TEM JEM-1220 (JEOL, Tokyo, Japan) at 80 kV and a TEM CCD Morada 11 megapixels camera (Olympus Soft Imaging Solutions, Munster, Germany) (Fig. 9). GO flakes formed 1–3 layers and they were angular, with rather sharp edges. The size in diameter of single flakes was from 2.1 to 4.5 μm. The Fourier transform infrared spectroscopy analysis, carried out by [23], showed graphene-specific peaks as well as the presence of –OH and C=O groups.

To prepare a nanofilm at the bottom of the culture plate, an aqueous solution of GO (100 mg/L) was used. Preparation of the nanofilm for culture consisted of covering the surface of the cell culture vessel with a thin layer, in an amount covering the entire surface (1 mL). Next, the GO was dried at room temperature under the laminar chamber, until thin, strongly sticking nanofilm of GO in each well were obtained. Then, the nGO surface was washed twice with sterile PBS to remove unattached GO flakes. The nGO surface was analysed with AFM method (Nanosurf, Liestal, Switzerland) and compared with the clear culture plate surface. The measurements included the maximum height of the roughness, maximum roughness valley depth, average roughness and root mean square roughness. The prepared plates with nGO were used for further experiments. The choice of 100 mg/L aqueous GO concentration was due to two reasons, namely; the cells willingly colonised the surface formed from the solution, as well as the images from TEM, which were formed from spotting on the formulated microscopic grids of an aqueous solution of GO at a concentration of 100 mg/L, pointed to a well-developed, thin layer of GO flakes.

To observe the hydrophilicity of nGO vs. plastic bottom of the culture vessel, 3 drops each of 10 µL clean water and 3 drops of 10 µL Dulbecco’s modified Eagle’s culture medium (DMEM; Thermo Fisher Scientific, Waltham, MA, USA) were gently pipetted onto the surface of the nGO and the clear culture dish (*n* = 6 in each group). After 5 min, the drops were captured using camera Canon EOS 5D and the circumference of the drops was measured with ImageJ software (Research Services Branch, National Institute of Mental Health, Bethesda, USA) [50], assuming that the increased hydrophilicity was proportional to the spread of the droplet. Briefly, in the first step, we calibrated the scale three times based on a digital photo, and the accuracy was 0.5 mm. Then we used the colour threshold tools. Finally, the droplet diameters were calculated automatically using the analyse particle tool.

Culture vessels with a GO dot pattern (Fig. 2a) were prepared for nGO biocompatibility testing. The drops (10 μL) of GO solution with standard concentration (100 mg/L) were applied to the bottom of the culture plate with a sterile pipette. After drying at room temperature under the laminar chamber, a pattern of dots was established, which was used to observe the potential colonisation of the seeded chicken mesenchymal stem cells, with a light optical inverted microscope (TL-LED, Leica Microsystems, Germany).

L-Glu was obtained from Merck Millipore (Darmstadt, Germany) and diluted with ultra-pure water to the concentration of 1000 mg/L. A solution of L-Glu was added to DMEM in an amount of 2% of the medium, therefore the concentration of L-Glu in the culture medium was 20 mg/L. In a preliminary study, the highest non-toxic concentration of L-Glu in the culture medium was determined. It was measured by the dose–response test, with the various L-Glu concentrations in the culture medium (from 1 to 100 mg/L). The positive and non-toxic effect of L-Glu on the number of viable cells was determined at the level of 20 mg/L. Therefore, this concentration (20 mg/L) of L-Glu was used in the described experiment.

### Experimental design

The experimental model was mesenchymal stem cells, collected from the hind limb of chicken embryo (Ross 308), purchased from a certificated hatchery. On the 8th day of embryogenesis, after incubation in standard conditions (37 °C and ~ 60% relative humidity) the embryos were sterile removed from the eggs. Next, the femoral tissue was precisely collected from the embryo’s hind limbs and gently placed in the solution of trypsin and incubated at the + 4 °C in a refrigerator for a 24 h. The next day, the activity of trypsin was neutralised by adding an equal volume of DMEM, and the sample was disintegrated by gentle pipetting through the tips. The concentration of living cells was measured with an automatic cell counter (NanoEnTek, Waltham, USA). The cells were seeded in flasks or dishes according to particular experiments in amount of 1.5 × 10^5^ viable cells/175 cm^2^, and culture in the medium was supplemented with 10% foetal bovine serum (Life Technologies, Houston, TX, USA) and 1% penicillin/streptomycin (Life Technologies, Houston, TX, USA) at 37 °C in a humidified atmosphere of 5% CO_2_/95% air in an Memmert ICO150med Incubator (Memmert, Schwabach, Germany).

Cells were divided into 4 groups; the control group and groups treated with nGO, L-Glu (2% of the medium) and nGOxL-Glu (2% of the medium). The culture medium was changed every second day.

### Cell morphology

Cell morphology was visualised with a light optical inverted microscope (TL-LED, Leica Microsystems, Germany) and a scanning electron microscope (SEM; Quanta 200, FEI, Hillsboro, USA), on day 5th of primary cell culture on the 6-well plates. For light microscope observation, cells were fixed in ice-cold methanol for 10 min, and then eosin/hematoxylin stained, according to the standard protocol. In turn, cells imaged in SEM were first fixed in 2.5% l-glutaraldehyde in phosphate-buffer saline (PBS; Life Technologies, Houston, USA), subsequently contrasted with osmium tetroxide (Sigma-Aldrich, St Louis, USA) and carbohydrazide (Sigma-Aldrich, St Louis, USA). Next, the cells were dehydrated in hexylene glycol (Sigma-Aldrich, St Louis, USA). Finally, drying was performed using a Polaron CPD 750 l critical point dryer (Quorum Technologies, Laughton, UK).

### Cell differentiation

The differentiation status of the primary muscle cells was evaluated after fluorescence staining assay, with the confocal microscope (Olympus FV1000, Tokyo, Japan). After 5 days of culture, cells were fixed in 4% paraformaldehyde in PBS (Life Technologies, Houston, USA; 10 min, RT), and washed with ice-cold PBS. Next, the cells on slides were permeabilised in Triton X-100 (Sigma-Aldrich, St Louis, USA) and washed with PBS (Sigma-Aldrich, St Louis, USA). Finally, cell nuclei were stained with 4′,6-diamidino-2-phenylindole (DAPI; Thermo Fisher Scientific, Waltham, USA) and actin filaments with phalloidin-Atto 633 (Sigma-Aldrich, St Louis, USA). After staining, the cells were washed with PBS, and slides were mounted under Fluoromount-G medium (Southern Biotech, Birmingham, USA). On the stained slides, the fusion index was calculated as a ratio of nuclei in the multinucleated myotubes (more than 2 myonuclei) to the total number of nuclei (mononucleated and multinucleated) [51, 52] in the randomly selected visual fields (*n* = 8). Next, the relative to control fusion index was calculated from the obtained results, as a ratio of the fusion index, for each group, compared to the control results. The nuclei were counted manually.

### Cell viability

Cell viability status was measured with the colorimetric MTT assay kit (ab211091, Abcam, Cambridge, MA, USA), according to the manufacturer’s protocol. Briefly, cells from the eighth-day chicken embryo femoral muscle were seeded into 96-well plate (Nest Scientific, Rahway, NJ, USA) at a density of 15 × 10^3^ in 100 μL of medium per well in eight replicates per each group treatment. Then, cells were incubated with experimental factors (L-Glu, nGO) for 48 h, in standard conditions (at 37 °C in a humidified atmosphere containing 5% CO_2_). After that time, the growth medium was removed from the wells and cells were incubated with 3-(4,5-dimethylthiazol-2-yl)-2,5-diphenyltetrazolium bromide (MTT) reagent for 3 h at 37 °C. After incubation, formazan crystals were dissolved in MTT solvent and incubated for 15 min. The absorbance of samples (*n* = 6) was measured at 570 nm on a microplate reader, Infinite^®^ 200 PRO microplate reader with i-control™ software (Tecan Group Ltd., Männedorf, Germany). Calculations were performed as described by Strojny et al. [53].

### Relative gene expression assay

Relative gene expression at the mRNA level was determined with quantitative real-time polymerase chain reaction (qPCR) method. The cells were obtained after 5 days of primary cell culture with experimental factors, by trypsinisation and centrifugation (1200 rpm for 5 min). The cell pellet was homogenised using a TissueLyser ball mill (Qiagen, Germantown, USA). Total RNA was extracted from cells and purified using a NucleoSpin^®^ RNA Plus XS (Macherey–Nagel GmbH and Co., Duren, Germany) with on-column DNase treatment, in accordance with the manufacturer’s recommendations. Total RNA concentration and purity were estimated using a NanoDrop ND-1000 spectrophotometer (Thermo Fisher Scientific, Waltham, MA, USA). 1 µg of RNA was used in the reaction of reverse transcription in a total volume of 20 µl using a Maxima first-strand cDNA synthesis kit (Thermo Fisher Scientific, Waltham, MA, USA) according to the manufacturer’s protocol. qPCR analyses were performed using a PowerUp SYBR green master mix (AppliedBiosystems, Foster City, USA). The reactions were run on an ABI Prism 7500 sequence detection system thermocycler (Applied Biosystems, Foster City, USA), according to the manufacturer’s recommended protocol.

Pairs of primers specific for the reference genes; *ACTB* and *GAPDH*, as well as for target genes, genes related to proliferation; *ATP5B*, *FGF2*, *LDHA*, *PCNA*, and genes related to differentiation; *MYF5*, *MYOD1*, *MYOG*, *PAX3* and P*AX7* were designed to span over intron sequences using PRIMER3 open-source software [54]. All primer data are listed in Table 2. The reference genes were used as the internal control to verify the quantitative real-time PCR. The 2-ΔΔCT method was used to determine the results.

**Table 2:** Primer sequences designed for quantitative real-time PCR analyses

Gene name	Primer sequence (5′→3′)		T. m. (^o^C)	Product size (bp)
Actin beta *(ACTB)*	F	GTCCACCTTCCAGCAGATGT	60.1	169
	R	ATAAAGCCATGCCAATCTCG	60.1	
Glyceraldehyde-3-phosphate dehydrogenase *(GAPDH)*	F	GCTAAGGCTGTGGGGAAAGT	60.5	161
	R	TCAGCAGCAGCCTTCACTAC	59.4	
ATP synthase, H+ transporting, mitochondrial F1 complex, beta polypeptide *(ATP5B)*	F	GTTATTCGGTGTTCGCTGGT	60.0	122
	R	GTAGACCAGAGCGACCTTGG	59.9	
Fibroblast growth factor 2 *(FGF2)*	F	GGCACTGAAATGTGCAACAG	60.3	151
	R	TCCAGGTCCAGTTTTTGGTC	59.9	
Lactate dehydrogenase A *(LDHA)*	F	CATGCCCACAACAAGATCAG	60.1	128
	R	CCTTTCAGCTTGTCCTCCAC	59.8	
Proliferating cell nuclear antigen *(PCNA)*	F	TGCACGCATTTGTAGAGACC	59.9	187
	R	AGTCAGCTGGACTGGCTCAT	60.0	
Myogenic factor 5 *(MYF5)*	F	CCAGGAGCTCTTGAGGGAAC	61.3	196
	R	ACTCTGCTCCGTCGCGTA	60.9	
Myogenic differentiation 1 *(MYOD1)*	F	AGCTCTCGCAGGAGAAACAG	59.9	160
	R	CTGGAGGCAGTATGGGACAT	60.0	
Myogenin *(MYOG)*	F	GGCTGAAGAAGGTGAACGAA	60.4	149
	R	CTGCTGGTTGAGGCTGCT	60.3	
Paired box 3 *(PAX3)*	F	CCGTGCTAGATGGAGGAAGC	61.9	157
	R	AGACACGGCTTGCGGTATG	61.9	
Paired box 7 *(PAX7)*	F	CAGTAGAGACAGGCCAAGC	59.2	134
	R	GGAGTTGGGAAGGAGTAGGG	59.9	

### Relative protein expression assay

Relative proteins expression was determined with Western blot analysis. At the 5th day of culture, the cells were detached with trypsin and centrifuged at 1200 rpm for 5 min. Whole-cell protein extracts were prepared, as described by Sosnowska et al. [55], using an ice-cold radioimmunoprecipitation assay. Next, total protein concentration in all samples was quantified using a bicinchoninic acid kit (Sigma-Aldrich, St. Louis, MO, USA). Tissue protein extracts, with concentrations of 40 µg (equal amounts of protein from each sample), were mixed with denaturing solution to a total volume of 20 µL (4 × Laemmli sample buffer with 2-mercaptoethanol; Bio-Rad, CA, USA), denatured for 5 min, and finally electrophoresed in 10% polyacrylamide gels, at the standard conditions (100 mA, 100 V, for 2 h in Tris–glycine-sodium dodecyl sulphate buffer) (Mini-PROTEAN^®^ Tetra vertical electrophoresis, Bio-Rad, CA, USA). At the next stage, proteins were electrotransferred onto Hybond 0.2 µm PVDF membrane (Amersham, GE Healthcare Bio-Sciences, MA, USA), with a Trans-blot turbo transfer system (Bio-Rad Laboratories, Munich, Germany). After blocking in 5% nonfat milk (Bio-Rad Laboratories, Munich, Germany) in PBS for 60 min, membranes were incubated overnight (at + 4 °C) with the selected primary antibodies: goat anti-PAX3 antibody (ab15717, Abcam, Cambridge, UK), mouse Anti-PAX7 antibody (ab199010, Abcam, Cambridge, UK), and rabbit to MYF5 antibody (ab139523, Abcam, Cambridge, UK).

Next, membranes were washed in cold PBS, and incubated with matched secondary antibodies: goat anti-mouse IgG, IgM (H + L) AP (T2192, Thermo Fisher Scientific, Waltham, MA, USA) or goat anti-rabbit IgG (H + L) AP (T1048, Thermo Fisher Scientific, Waltham, MA, USA) or donkey anti-goat IgG (H + L) AP (A16002, Thermo Fisher Scientific, Waltham, MA, USA) for 1 h, at RT. Visualisation of membranes was performed using the Western-Star™ immunodetection system (No. T1046, Applied Biosystems, Foster City, USA) in Azure c400 apparatus (Azure Biosystems, Dublin, CA, USA). Next, the membranes were stripped with Restore Western blot stripping buffer (No. 21059, Thermo Scientific, Waltham, MA, USA). All procedures were performed again with loading control primary antibody: actin beta antibody (ACTB; MA5-15739, Thermo Fisher Scientific, Waltham, MA, USA). Densitometric analysis of the relative protein expression was performed using ImageJ software (Research Services Branch, National Institute of Mental Health, Bethesda, USA) [50, 56].

### Statistical analysis

Data were analysed by one-way ANOVA. The differences in-between groups were evaluated by t-test (Fig. 2b) or Tukey´s HSD test (others results). Statistical analysis was performed using the IBM SPSS statistics ver. 25 (SPSS Inc. Chicago, IL, USA). Data are expressed as the mean and standard error of mean. For all tests, statistical differences with *p* ≤ 0.05 were considered significant.

## Data Availability

The datasets used and/or analysed during the current study are available from the corresponding author on reasonable request.

## Abbreviations

ACTB: Actin beta
AFM: Atomic force microscopy
ANOVA: Analysis of variance
ATP5B: ATP synthase, H+ transporting, mitochondrial F1 complex, beta polypeptide
DAPI: 4′,6-Diamidino-2-phenylindole
DMEM: Dulbecco’s modified Eagle medium
ECM: Extracellular matrix
FGF2: Fibroblast growth factor 2
GAPDH: Glyceraldehyde-3-phosphate dehydrogenase
GO: Graphene oxide
hASCs: Human adipose stem cells
LDHA: Lactate dehydrogenase A
L-Glu: l-glutamine
MAPK: Mitogen-activated protein kinase
MTT: 3-(4,5-Dimethylthiazol-2-yl)-2,5-diphenyltetrazolium bromide
MYF5: Myogenic factor 5
MYOD1: Myogenic differentiation 1
MYOG: Myogenin
nGO: Graphene oxide nanolayer
PAX3: Paired box 3
PAX7: Paired box 7
PCNA: Proliferating cell nuclear antigen
rGO: Reduced graphene oxide
SEM: Scanning Electron Microscope
TNF-α: Tumor necrosis factor alpha
